# Combining Human Umbilical Cord Blood Cells With Erythropoietin Enhances Angiogenesis/Neurogenesis and Behavioral Recovery After Stroke

**DOI:** 10.3389/fneur.2019.00357

**Published:** 2019-04-10

**Authors:** Sunyoung Hwang, JeeIn Choi, MinYoung Kim

**Affiliations:** ^1^Rehabilitation and Regeneration Research Center, CHA University, Seongnam, South Korea; ^2^Department of Rehabilitation Medicine, CHA Bundang Medical Center, College of Medicine, CHA University, Seongnam, South Korea

**Keywords:** stroke, human umbilical cord blood cell, erythropoietin, functional recovery, neurogenesis, angiogenesis

## Abstract

Disruption of blood flow in the brain induces stroke, the leading cause of death and disability worldwide. However, so far the therapeutic options are limited. Thus, the therapeutic efficacy of cell-based approaches has been investigated to develop a potential strategy to overcome stroke-induced disability. Human umbilical cord blood cells (hUCBCs) and erythropoietin (EPO) both have angiogenic and neurogenic properties in the injured brain, and their combined administration may exert synergistic effects during neurological recovery following stroke. We investigated the therapeutic potential of hUCBC and EPO combination treatment by comparing its efficacy to those of hUCBC and EPO alone. Adult male Sprague-Dawley rats underwent transient middle cerebral artery occlusion (MCAO). Experimental groups were as follows: saline (injected once with saline 7 d after MCAO); hUCBC (1.2 × 10^7^ total nucleated cells, injected once via the tail vein 7 d after MCAO); EPO (500 IU/kg, injected intraperitoneally for five consecutive days from 7 d after MCAO); and combination of hUCBC and EPO (hUCBC+EPO). Behavioral measures (Modified Neurological Severity Score [mNSS] and cylinder test) were recorded to assess neurological outcomes. Four weeks after MCAO, brains were harvested to analyze the status of neurogenesis and angiogenesis. *In vitro* assays were also conducted using neural stem and endothelial cells in the oxygen-glucose deprivation condition. Performance on the mNSS and cylinder test showed the most improvement in the hUCBC+EPO group, while hUCBC- and EPO-alone treatments showed superior outcomes relative to the saline group. Neurogenesis and angiogenesis in the cortical region was the most enhanced in the hUCBC+EPO group, while the findings in the hUCBC and EPO treatment alone groups were better than those in the saline group. Astrogliosis in the brain tissue was reduced by hUCBC and EPO treatment. The reduction was largest in the hUCBC+EPO group. These results were consistent with *in vitro* assessments that showed the strongest neurogenic and angiogenic effect with hUCBC+EPO treatment. This study demonstrates that combination therapy is more effective than single therapy with either hUCBC or EPO for neurological recovery from subacute stroke. The common pathway underlying hUCBC and EPO treatment requires further study.

## Introduction

Stroke, caused by the disruption of cerebral blood flow, is a leading cause of death and major disability throughout the world (1). However, the therapeutic options to deal with stroke are limited. Despite efforts to develop new therapies for stroke, all treatments have thus far failed to show a clinical effect or are known to have potential toxic effects. Although intra-arterial thrombolysis and intravenous tissue plasminogen activator therapy have been developed and used for ischemic stroke as effective approaches, those are actually significant only in acute phase with risk of cerebral hemorrhage (1–3). After the onset of stroke, patients experience the greatest amount of neurological recovery during the 3 months post-stroke, which does not last afterwards (4). Before fixation of the impairment in the chronic stage, subacute stroke patients require an effective therapeutic measure, which has remained elusive to date.

While many drugs from successful preclinical experiments have failed in clinical trials for stroke (5), cell therapy has been introduced and expected to be effective by ameliorating neurological impairments due to stroke *in vivo* with relevant mechanisms identified *in vitro* (6–8). Although cell-based therapy has therapeutic potential, to date, the greatest limitation must be the lack of clear evidence related to efficacy (9, 10) and safety issues that limit active clinical trials (10).

Human umbilical cord blood cells (hUCBCs) are a rich source of various progenitor cells, including hematopoietic stem cells that can be used as a cell therapy agent and are known to be safe based on 30 years of clinical application (7, 11). The therapeutic efficacy of hUCBC was supported by significant neurological recovery based on modified Neurological Severity Scores (mNSS) for both acute and subacute brain injury (7). Furthermore, evidence on neurogenesis and angiogenesis, in addition to functional recovery, in a stroke model was observed following hUCBC transplantation (12). Our previous clinical study in children with cerebral palsy also revealed functional improvements following hUCBC administration (13).

As a potential approach to enhance the therapeutic potency of cell therapy, combination therapy with a growth factor can be used (14). In this study, erythropoietin (EPO) was selected among the candidate molecules because it has been used clinically as a safe drug. EPO is a member of the hematopoietic cytokine superfamily and has neuroprotective effects against ischemic brain insults (15, 16). Repeated pre-treatment with EPO produced a neuroprotective effect in both focal and global ischemia models (15, 17, 18). Furthermore, EPO enhances angiogenesis and neurogenesis after ischemic stroke, leading to accompanying functional recovery (19–21). Therefore, EPO may be a promising therapeutic agent to enhance hUCBC treatment in ischemic stroke to induce neurogenesis and angiogenesis.

In recovery from stroke, not only neurogenesis, but also coupled angiogenesis, play central roles (22). For example, treatment with human bone marrow stromal cells (hBMSCs) has been found to enhance angiogenesis in the ischemic boundary zone after stroke (23). Moreover, combination treatment with simvastatin, sodium ferulate, and n-butylidenephthalide following hBMSC administration induced neurological improvement with findings of neurogenesis, angiogenesis, and arteriogenesis after cerebral ischemia (24, 25).

Therefore, in this study, we used a well-established rat model of transient ischemia (26) to represent subacute stroke to analyze the therapeutic efficacy of either hUCBC or EPO, and the additive effects of concomitant treatment with both. In addition, we have systematically investigated the underlying mechanism of neurogenesis and angiogenesis not only in *in vivo* but also *in vitro* experiments.

## Materials and Methods

### Preparation of Human Umbilical Cord Blood Cells

Human UCB containing citrate phosphate dextrose adenine as an anticoagulant was provided from the cord blood bank of CHA Medical Center. Informed by previous clinical trials (27), we injected a total of 3 × 10^7^ kg nucleated cells. This experiment was approved by the Institutional Ethics Committee of CHA Bundang Medical Center, Korea.

### Animals

Male Sprague-Dawley rats (260–300 g; Charles River Laboratories, South Korea) were acclimated to their environments for 2 days before use. The rats were housed in a temperature-controlled room (22 ± 2°C), kept at constant humidity (50 ± 10%), and maintained on a 12-h light/dark cycle with *ad libitum* access to food and water. The provision of food was stopped from the night before surgery until the day of surgery, but water was continuously supplied (26). Neural stem cells (NSCs) were isolated from 1-to 3-day-old ICR mice (Charles River Laboratories, South Korea). All experimental procedures involving animals were performed in accordance with the Guide for the Care and Use of Laboratory Animals as adopted and promulgated by the U.S. National Institutes of Health and were approved by CHA University Institutional Animal Care & Use Committee (IACUC150018, IACUC180018, IACUC180181).

### *In vivo* Ischemia Model: Transient Middle Cerebral Artery Occlusion (MCAO)

Rats were initially anesthetized with 3.0% isoflurane in 70% N_2_O and 30% O_2_ (v/v) mixture supplied via a facemask and maintained with 2.5% isoflurane. Briefly, to occlude the middle cerebral artery (MCA), a 4-0 monofilament nylon suture (Ethicon Johnson & Johnson, Brussels, Belgium) with a heat-blunted end was inserted into the right external carotid artery and advanced into the internal carotid artery.

After a midline incision was made, the right MCA was occluded for 90 min as previously described. It was then reperfused by gentle removal of the suture. Body temperature was maintained at 37°C with a homeothermic blanket and checked using a rectal temperature probe.

### Treatment and Experimental Groups

The number of total nucleated cells of hUCBC used in treatment was based on our previous clinical trial which was performed in patients with traumatic brain injury (27). Furthermore, the dosage of EPO was selected based on a corresponding dose in a clinical trial for cerebral palsy (28) that is considered safe for humans.

A total of adult male Sprague–Dawley rats were randomly divided into four groups: (1) saline group (injected into the tail vein once at 7 d post-MCAO / intraperitoneal injection for five consecutive days from 7 d post-MCAO); (2) EPO group (EPO, 500 IU/kg, intraperitoneal injection for 5 consecutive days from 7 d post-MCAO); EPO set included saline (*n* = 6) and EPO (*n* = 5) groups; (3) hUCBC group (hUCBC, 1.2 × 10^7^ total nucleated cells, tail vein injection once at 7 d post-MCAO; hUCBC set included saline (*n* = 9) and hUCBC (*n* = 6) groups; (4) hUCBC+EPO group (hUCBC+EPO treatment at the same dose and schedule as the other groups; hUCBC+EPO set included saline (*n* = 6) and hUCBC+EPO (*n* = 5) groups. At 7 d post-MCAO, behavioral studies were performed immediately before injections.

### Behavioral Tests to Assess Neurological Function

The mNSS is a composite scale assessing motor function, sensory disturbance, reflex, and balance tests. Table 1 shows mNSS scores obtained in this study (7, 29). Neurological function was graded on a scale of 0–18 (normal score: 0; maximal deficit score: 18). Only rats with 11–13 points on the mNSS 1 day post-reperfusion were used in this study. Overall exclusion rate due to score criteria at 1 d post-MCAO was 20%, and survival rate to the end of the experiment was 62.5% among the included subjects.

**Table 1:** Modified Neurological Severity Score (mNSS).

	**Points**
Motor Tests	
Raising rat by the tail 1 Flexion of forelimb 1 Flexion of hindlimb 1 Head moved > 10° to vertical axis within 30 s	3
Placing rat on the floor (normal = 0; maximum = 3) 1 Normal walk 1 Inability to walk straight 1 Circling toward the paretic side 1 Fall down to the paretic side	3
Sensory tests 1 Placing test (visual and tactile test) 1 Proprioceptive test (deep sensation, pushing the paw against the table edge to stimulate limb muscles)	2
Beam balance tests (normal = 0; maximum = 6) 1 Balances with steady posture 1 Grasps side of beam 1 Hugs the beam and one limb falls down from the beam 1 Hugs the beam and two limbs fall down from the beam, or spins on beam(>60 s) 1 Attempts to balance on the beam but falls off (>40 s) 1 Attempts to balance on the beam but falls off (>20 s) 1 Falls off: no attempt to balance or hang on to the beam (< 20 s)	6
Reflexes absent and abnormal movements 1 Pinna reflex (head shake when touching the auditory meatus) 1 Corneal reflex (eye blink when lightly touching the cornea with cotton) 1 Startle reflex (motor response to a brief noise from snapping a clipboard paper) 1 Seizures, myoclonus, myodystony	4
Maximum points	18

*One point is awarded for the inability to perform the tasks or for the lack of a tested reflex; 13–18 indicates severe injury; 7–12, moderate injury; 1–6, mild injury*.

The cylinder test was performed to assess the degree of forepaw asymmetry. Rats were placed in a transparent Plexiglas cylinder (diameter: 20 cm, height: 30 cm), and the number of forepaw contacts with the cylinder wall was counted until a total of 20 contacts was reached (30). The study team has previously established the reliabilities (26) of the mNSS and cylinder tests, which were performed at 0, 1, 3, 7, 14, 21, and 28 days after MCAO.

### Gross Finding of Brain Section With Cresyl Violet Staining

Serial 8-μm coronal brain sections were prepared using a cryostat and stained with 0.5% cresyl violet. Image of each cresyl violet-stained brain slice was obtained using a light microscope (Nikon, USA).

### Immunofluorescence Staining

At 28 d after MCAO (21 d post-therapy), animals were anesthetized with 3.0% isoflurane in 70% N_2_O and 30% O_2_ (v/v) and then perfused transcardially with a freshly prepared solution of 4% paraformaldehyde in phosphate-buffered saline (PBS). The brains were removed and post-fixed overnight in the same fixative before being immersed in a solution of 30% sucrose in PBS. Serial 8-μm-thick coronal tissue sections were cut using a microtome and immunostained as free-floating sections. The sectioned brain tissues were washed in PBS and incubated for 1 h in blocking solution (2% normal goat serum, 0.5% Triton^TM^ X-100 in PBS). The fluorescent target was selected according to the desired purpose; Sox2 was used to assess neuronal stem cell, Ki67 as a proliferation marker, NeuN to assess neuronal survival, glial fibrillary acidic protein (GFAP) to reveal astrogliosis status, CD31, which is also known as PECAM-1, to assess endothelial expression, and VEGF as an angiogenic marker. The brain tissues were incubated overnight at 4°C with the following primary antibodies: anti-Sox2 (1:500, Santa Cruz, USA), anti-Ki67 (1:500, Abcam, UK), anti-NeuN (1:1000, Novus, USA), anti-GFAP (1:1000, Abcam, UK), anti-CD31 (1:100, Abcam, UK), and anti-VEGF (1:100, Novus, USA). After rinsing with primary antibodies, the tissues were incubated with secondary antibodies for 1 h at 22–25°C. The brain tissues with DAPI (Molecular Probes, Invitrogen, USA) mounting solution were imaged using a fluorescence microscope (Nikon, USA).

### Primary Neural Stem Cell Culture

Neural stem cells (NSCs) were isolated from 1-to 3-day-old ICR mice (Charles River Laboratories, South Korea). The whole cortex of the mouse brain was dissected and cultured with basal medium, including Dulbecco's Modified Eagle Medium: Nutrient Mixture F-12 (DMEM/F12; Gibco, USA), 2% B-27 supplement (Gibco, USA), 1 × GlutaMAX (Gibco, USA), basic fibroblast growth factor (bFGF, 20 ng/ml; Gibco, USA), and epidermal growth factor (EGF, 10 ng/ml; Gibco, USA). NSCs were maintained at 37°C in a 5% CO_2_ incubator and were subcultured when 90% confluence was reached following a 3-day interval.

### Mouse bEnd.3 Cell Culture

bEnd.3 mouse brain endothelial cells were purchased from the ATCC (Manassas, VA, USA) and cultured with medium, including DMEM (Gibco, USA), 10% fetal bovine serum (FBS; Gibco, USA), and 1% penicillin and streptomycin (Gibco, USA). bEnd.3 cells were subcultured when 90% confluence was reached using 0.25% trypsin EDTA and were maintained at 37°C in a 5% CO_2_ incubator.

### Oxygen-Glucose Deprivation and Cell Treatment

For hypoxic experiments in bEnd.3 cells and NSCs, cells were seeded in 6-well plates 5 × 10^5^cells per well and underwent oxygen-glucose deprivation (OGD) conditioning under 5% CO_2_ and 95% N_2_ at 37°C in the chamber for 24 h for bEnd.3 cells and 5 h for NSCs (31, 32). After OGD, we treated cells with EPO (0.5 IU/ml), co-cultured with hUCBCs (2 × 10^4^ total nucleated cells per well), or combined hUCBC co-culture and EPO treatment to confirm therapeutic effect in a transwell (0.4-μm pore size; SPL, South Korea), and analyzed 24 h after treatment.

### Cell Viability

Cell concentration was measured by cell counting after staining with trypan blue (STEMCELL, USA) using a cell counter (Thermo Fisher, USA). Cell viability was evaluated using a Cellrix viability assay kit (Cellrix, USA). Cells were cultured overnight at 1 × 10^3^ cells per well in 96-well plates. After adding 10 μl/per well of WST-8 into the cell media, we detected absorbance at 450 nm after 30 min incubation. In all viability assays, wells were assessed in triplicate for each group.

### Conventional Reverse Transcription Polymerase Chain Reaction (RT-PCR)

Total RNA was isolated from bEnd.3 cells and NSCs using TRIZOL (Life Technologies, USA) and cDNA was synthesized using a reverse transcriptase kit (Toyobo, Japan). Using Taq PCR Master mix (Bioneer, South Korea), PCR was performed. The levels of neuronal markers, Sox2 and Nestin (33), and angiogenesis markers, VEGF, and bFGF (34), were measured after administration of EPO, hUCBC, and hUCBC+EPO in OGD-induced cells. The primers for neuronal markers were Sox2 forward, 5′-CAATCCCATCCAAATTAACGCA-3′, Sox2 reverse, 5′- AAGCTGCAGAATCAAAACCC-3′, Nestin forward, 5′-ACCTATGTCTGAGGCTCCCTATCCTA-3′, and Nestin reverse, 5′-GAGGTTGGATCATCAGGGAAGTG-3′; for angiogenic markers, VEGF forward, 5′- CACAGCAGATGTGAATGCAG-3′, VEGF reverse, 5′-TTTACACGTCTGCGGATCTT-3′, bFGF forward, 5′-CAACCGGTACCTTGCTATGA-3′, and bFGF reverse, 5′-TCCGTGACCGGTAAGTATTG-3′. The following cycling conditions were used: 5 min at 95°C for polymerase activation, followed by 95°C for 30 s and 60°C for 30 s. This was repeated for 35 cycles, and findings were normalized to β-actin.

### Western Blotting

To confirm the difference in expressed proteins between the bEnd.3 cells and NSCs, proteins were extracted from ipsilesional brain tissue using a RIPA lysis buffer (Thermo Fisher, USA). For western blotting, equal amounts (30 μg) of protein extracts in a lysis buffer were used in a 12% SDS-PAGE analysis and transferred onto the polyvinylidene fluoride membranes. A 5% skimmed milk solution was used to block the membrane for 1 h at room temperature on a rocker. The housekeeping gene, β-actin, was employed as a loading control. Anti-Sox2 (1:1000, Santa Cruz, USA), anti-Nestin (1:1000, Abcam, UK), anti-VEGF (1:1000, Novus, USA), anti-bFGF (1:1000, Santa Cruz, USA), anti-CD31 (1:000, Abcam, UK), and anti-β-actin (1:1000, Santa Cruz, USA) antibodies were incubated with the membranes at 4°C overnight. After washing the membranes with TBST buffer, a horseradish peroxidase-conjugated anti-rabbit IgG antibody (Santa Cruz, USA) at a dilution of 1:20,000 or an anti-mouse IgG antibody (KPL, Inc., Gaithersburg, MD, USA) at a dilution of 1:10,000 was added to the corresponding primary antibodies, followed by incubation for 1 h at room temperature. Bands were detected using ECL reagent (Millipore, USA).

### Tube Formation

The tube formation assays were performed using 24-well plates coated with 200 μl of Matrigel (Gibco, USA) per well, which was incubated at 37°C for 30 min to solidify. Next, 1 × 10^5^ bEnd.3 cells were seeded per well in a coated plate. Tube formation was estimated after 8 h of cell culture. As a positive control, bFGF (20 ng/ml) was added to induce angiogenesis in endothelial cell (35). Three random areas in the cortex were imaged using a microscope (Nikon, USA), and the total number of tube structures was calculated.

### Statistical Analyses

Data are presented as the mean ± standard error of mean (*N* = 3–9 per group). Each experiment was repeated at least three times. Statistical comparisons between groups was performed using one-way ANOVA and Prism6.0 software (GraphPad Software, San Diego, CA, USA). *P* < 0.05 was considered statistically significant.

## Results

### EPO, hUCBC, and hUCBC+EPO Attenuated Ischemic Brain Damage

**Figure 1:**
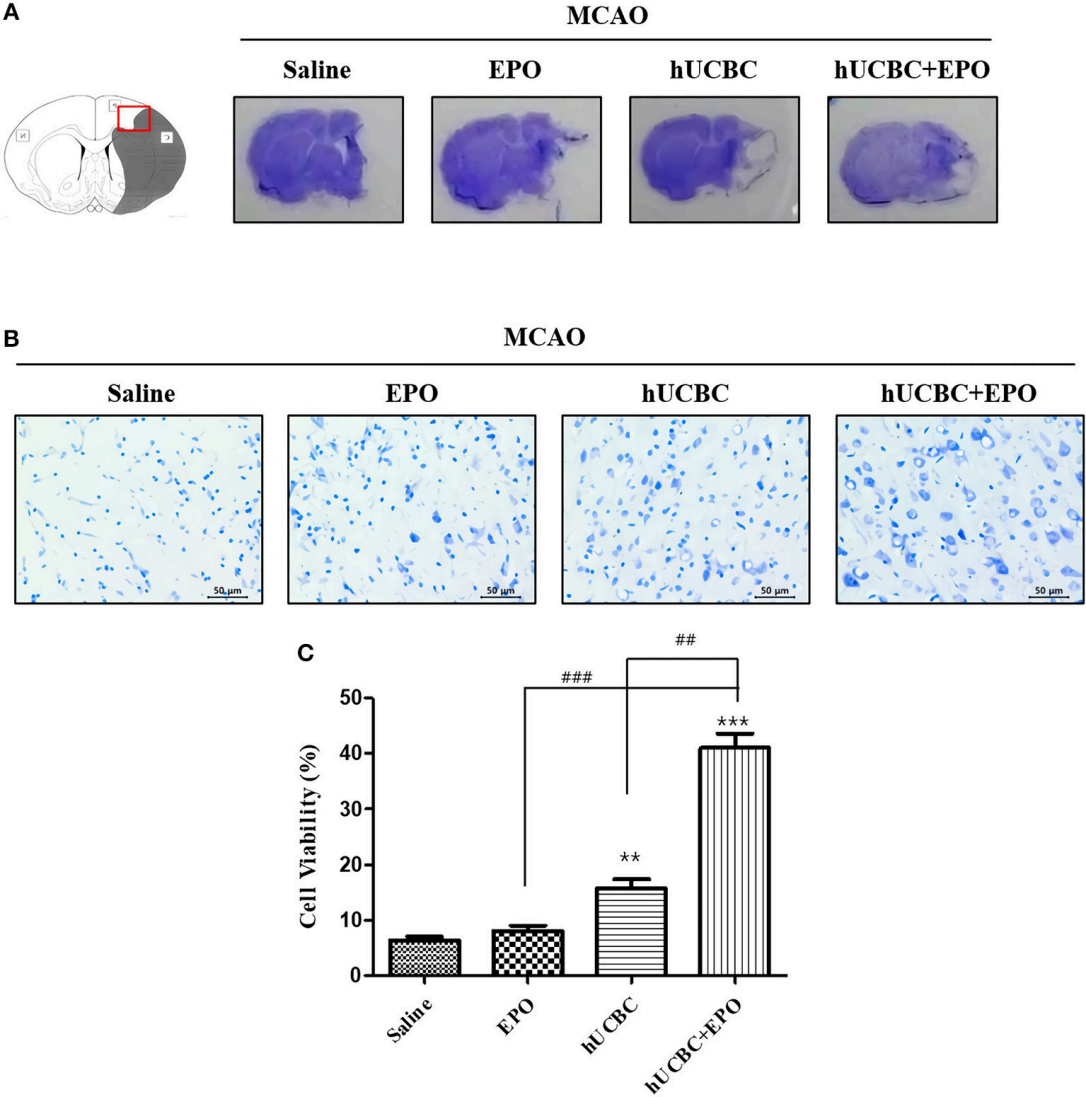
EPO, hUCBC, and hUCBC+EPO attenuated brain damage in MCAO. **(A)** Gross finding of brain section 28 d after MCAO. **(B)** Representative images of cresyl violet staining in the cortex (**B**, scale bar 50 μm). **(C)** Quantification of the number of neurons in the cortex. Data represent mean ± SEM. *N* = 3 per group. ^**^P < 0.01, ^***^P < 0.005 vs. Saline group. ^##^*P* < 0.01, ^###^*P* < 0.005 for inter-treatment group comparison.

We investigated whether EPO, hUCBC, and hUCBC+EPO affect neuronal damage after brain ischemia at 21 d post-therapy. Animals that received hUCBC+EPO exhibited the highest reduction of brain tissue volume loss; the hUCBC-alone group also exhibited more reduction of brain tissue volume loss than the saline group (Figure 1A).

Brains were histologically examined with cresyl violet staining to determine the neuroprotective effects of EPO, hUCBC, and hUCBC+EPO. Treatment with hUCBC+EPO achieved the most significant alleviation of cell damage in rat brains as evinced by the number of intact neurons (*Ps* < 0.01 vs. saline, EPO, hUCBC); the hUCBC- and EPO-alone groups also exhibited significant reductions in the number of injured and shrunken neurons compared to the saline group (*Ps* < 0.01 vs. saline) (Figures 1B,C).

### Therapeutic Efficacy of EPO, hUCBC, and hUCBC+EPO in the Subacute Stroke Model

**Figure 2:**
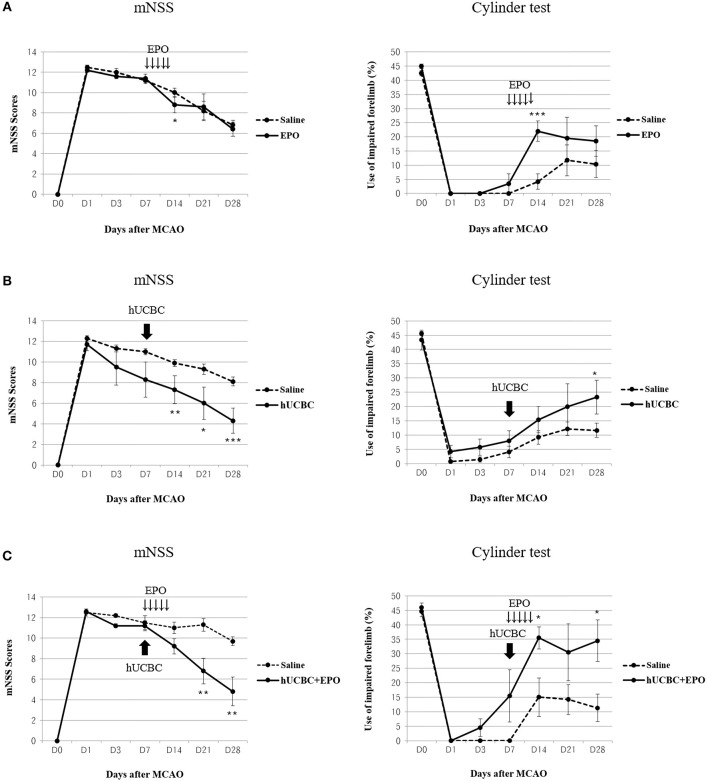
Behavior changes after EPO, hUCBC and hUCBC+EPO administration in comparison with saline treatment for each set of experiments in a rat model of middle cerebral artery occlusion (MCAO). Saline (MCAO alone); EPO (500 IU/kg, injected intraperitoneally for 5 consecutive days from 7 d after MACO); hUCBC (1.2 × 10^7^, injected via tail vein once at 7 d after MCAO); and combination of hUCBC and EPO (hUCBC+EPO). Data represent mean ± standard error of mean (SEM) of mNSS and Cylinder test scores **(A–C)**. *N* = 5–9 per group. ^*^P < 0.05, ^**^P < 0.01, ^***^P < 0.005 vs. Saline group.

Functional performance as assessed using the mNSS and cylinder test showed bigger improvement in the hUCBC+EPO group (*P* < 0.01 for mNSS at 14 d and 21 d post-therapy; *P* < 0.05 for cylinder test at 7 d and 21 d post-therapy) than the saline group (Figure 2C). Also, hUCBC-alone (*P* < 0.05 for mNSS at 7 d, 14 d, and 21 d post therapy; *P* = 0.05 for cylinder test at 21 d post-therapy) and EPO-alone (*P* < 0.05 for mNSS at 7 d post therapy; *P* < 0.01 for cylinder test at 7 d post-therapy) groups showed bigger improvements than the saline group (Figures 2A,B). Improvement following EPO treatment was significant only at 7 d post-therapy (Figure 2A). On the contrary, the efficacy of hUCBC administration was most prominent at 21 d post-therapy (Figure 2B). Only improvement in the hUCBC+EPO group was apparent from 7 d post-therapy and continued to 21 d post-therapy and the amount of improvement was biggest compared to the EPO-alone and hUCBC alone groups (Figure 2C) although the efficacy tests were conducted for each pair of the intervention with saline group.

### EPO, hUCBC, and hUCBC+EPO Increased the Proliferation of Neuronal Cells in the Subacute Stroke Model

**Figure 3:**
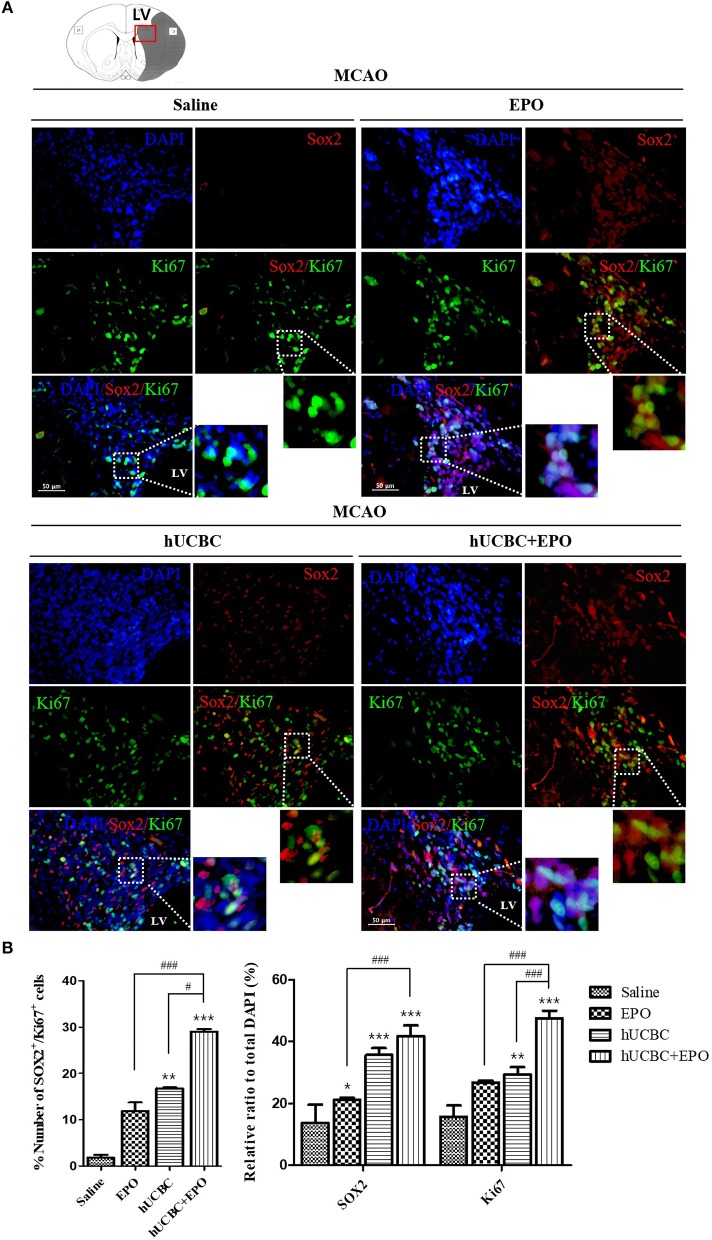
Immunohistochemistry findings in subventricular zone of lateral ventricle following EPO, hUCBC and hUCBC+EPO treatments which administered at or from 7 d after MCAO. Coronal brain slices were stained with Ki67 or Sox2 antibodies at 28 d after MCAO. **(A)** The co-localization of Sox2 (Red) and Ki67 (Green) identifies proliferation of neuronal cells in the lateral ventricle. White boxes in images indicate the zone of co-localization for further zoomed images. Representative images (scale bar 50 μm). **(B)** Quantification of the number of Sox2(+) cells, Ki67(+) cells, and Sox(+)/Ki67(+) in the subventricular zone of lateral ventricle. Data represent mean ± SEM. *N* = 3 per group. ^*^P < 0.05, ^**^P < 0.01, ^***^P < 0.005 vs. Saline group. ^#^*P* < 0.05, ^###^*P* < 0.005 for inter-treatment group comparison.

The quantitative analysis of fluorescent immunostaining images of Sox2(+) and Ki67(+) in subventricular zone of lateral ventricle was conducted. The numbers of Sox2(+) cells, Ki67(+) cells, and Ki67/Sox2 double(+) cells in the subventricular zone were higher in the EPO- and hUCBC-alone groups and hUCBC+EPO group than in the saline group (Figures 3A,B). Co-localization of Ki67 and Sox2 was greatest in hUCBC+EPO group. This result indicates increment of proliferation of neuronal cells in the lateral subventricular zone by treatment with EPO, hUCBC, and hUCBC+EPO.

### EPO, hUCBC, and hUCBC+EPO Increased Neurogenesis and Reduced Inflammation in the Subacute Stroke Model

**Figure 4:**
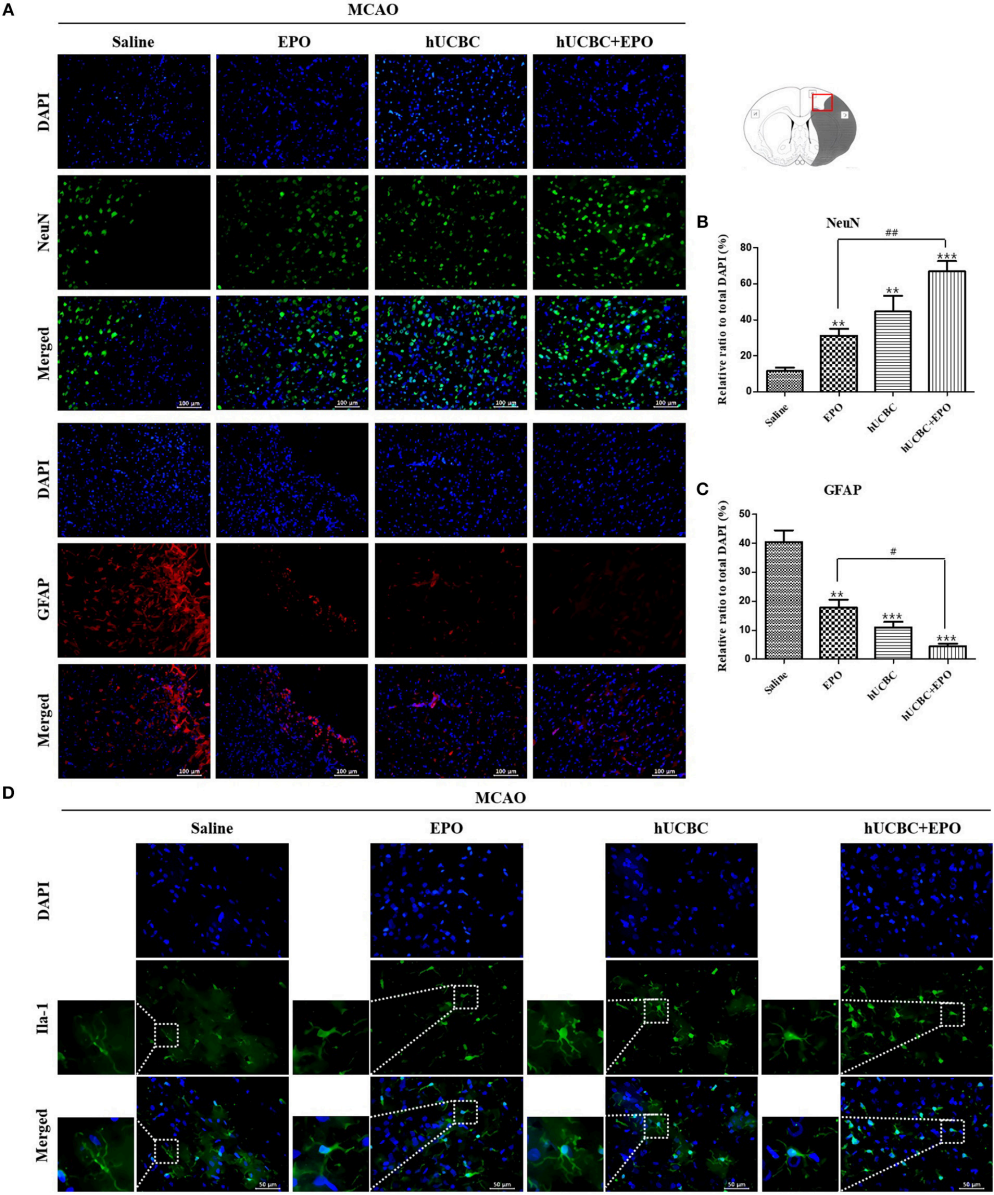
Immunohistochemistry findings in the cerebral cortex following EPO, hUCBC and hUCBC+EPO treatments which were given at or from 7 d after MCAO. Coronal brain slices were stained with anti-NeuN-, anti-GFAP-, or Iba-1 antibodies 28 d after MCAO. **(A)** Representative images [**(A)**, scale bar 100 μm]. **(B,C)** Quantification of the cell density of NeuN(+) and GFAP(+) cells in the cortex of ipsilesional are provided. The treatments promoted neuron, reduced astrogliosis, and changed microglia morphology in the cortex; the strongest findings were observed in the hUCBC+EPO group. **(D)** Representative images (**D**, scale bar 50 μm). Data represent mean ± SEM. *N* = 3 per group, ^**^P < 0.01, ^***^P < 0.005 vs. saline-treatment group. ^#^*P* < 0.05, ^##^*P* < 0.01 for inter-treatment group comparison.

To examine whether the administration of EPO, hUCBC, and hUCBC+EPO affected neurogenesis, NeuN(+) cells were counted, and astrogliosis was assessed by counting GFAP(+) cells in the cortex at 28 days after MCAO. Figures 4A,B show the significantly more NeuN(+) neuronal cells in the hUCBC+EPO group than in saline (*P* < 0.0001) or EPO-alone groups (*P* < 0.01). The hUCBC group featured the next greatest number of NeuN(+) neuronal cells, which was also higher than those in the saline and in EPO groups (*P* < 0.01).

The number of GFAP(+) cells were markedly reduced in the hUCBC+EPO (*P* < 0.001) group in the cortical region, while the EPO- (*P* < 0.01) and hUCBC- (*P* < 0.001) alone treatment groups also showed reductions in the number of GFAP(+) cells, although to a lesser extent (*P* < 0.05 for EPO vs. hUCBC+EPO). In contrast, astrogliosis was obvious in the saline group with many GFAP(+) cells present (Figures 4A,C).

More microglia survived at ischemic lesions in the EPO, hUCBC, and hUCBC+EPO groups than in the saline group, as evinced by the decrease in the number of dying Iba-1 (+) cells with fragmented cellular processes (Figure 4D).

### EPO, hUCBC, and hUCBC+EPO Enhanced Angiogenesis in the Subacute Stroke Model

**Figure 5:**
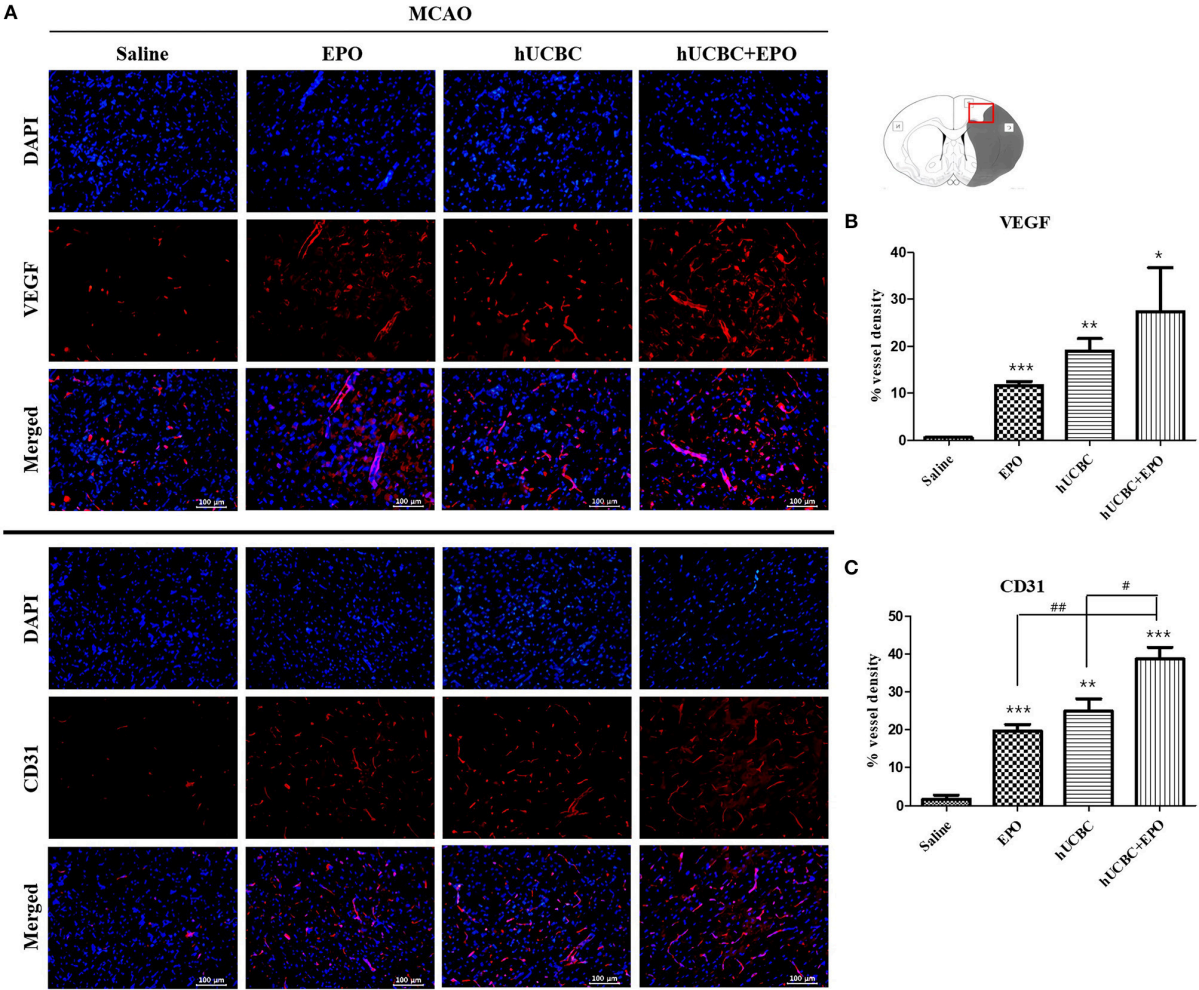
Immunohistochemistry findings in the cerebral cortex following EPO, hUCBC and hUCBC+EPO treatments which were given at or from 7 d after MCAO. Coronal brain slices were stained with anti-VEGF or anti-CD31 antibodies 28 d after MCAO. **(A)** Representative images (**A**, scale bar 100 μm). **(B,C)** Quantification for the vessel density of VEGF(+) and CD31(+) cells in the cortex, and the highest densities of cells were observed in hUCBC+EPO group. Data represent mean ± SEM. *N* = 3 per group. ^*^P < 0.05, ^**^P < 0.01, ^***^P < 0.005 vs. saline-treated group. ^#^*P* < 0.05, ^##^*P* < 0.01 for inter-treatment group comparison.

The quantitative analysis of fluorescent immunostaining images showed that hUCBC+EPO brains exhibited the greatest cortical vessel density of VEGF(+); cortical vessel density of VEGF(+) cells was higher in the EPO- and hUCBC-alone groups than the saline group (*P* < 0.05) (Figures 5A,B).

Similarly, the greatest vessel density of cells positive for CD31, a vascular endothelial marker, was observed in the hUCBC+EPO group; the EPO- and hUCBC-alone groups exhibited more vessel density expression of CD31(+) cells than did the saline group (*P* < 0.01) (Figures 5A,C).

### EPO, hUCBC, and hUCBC+EPO Enhanced Neurogenesis in OGD-Injured NSC

**Figure 6:**
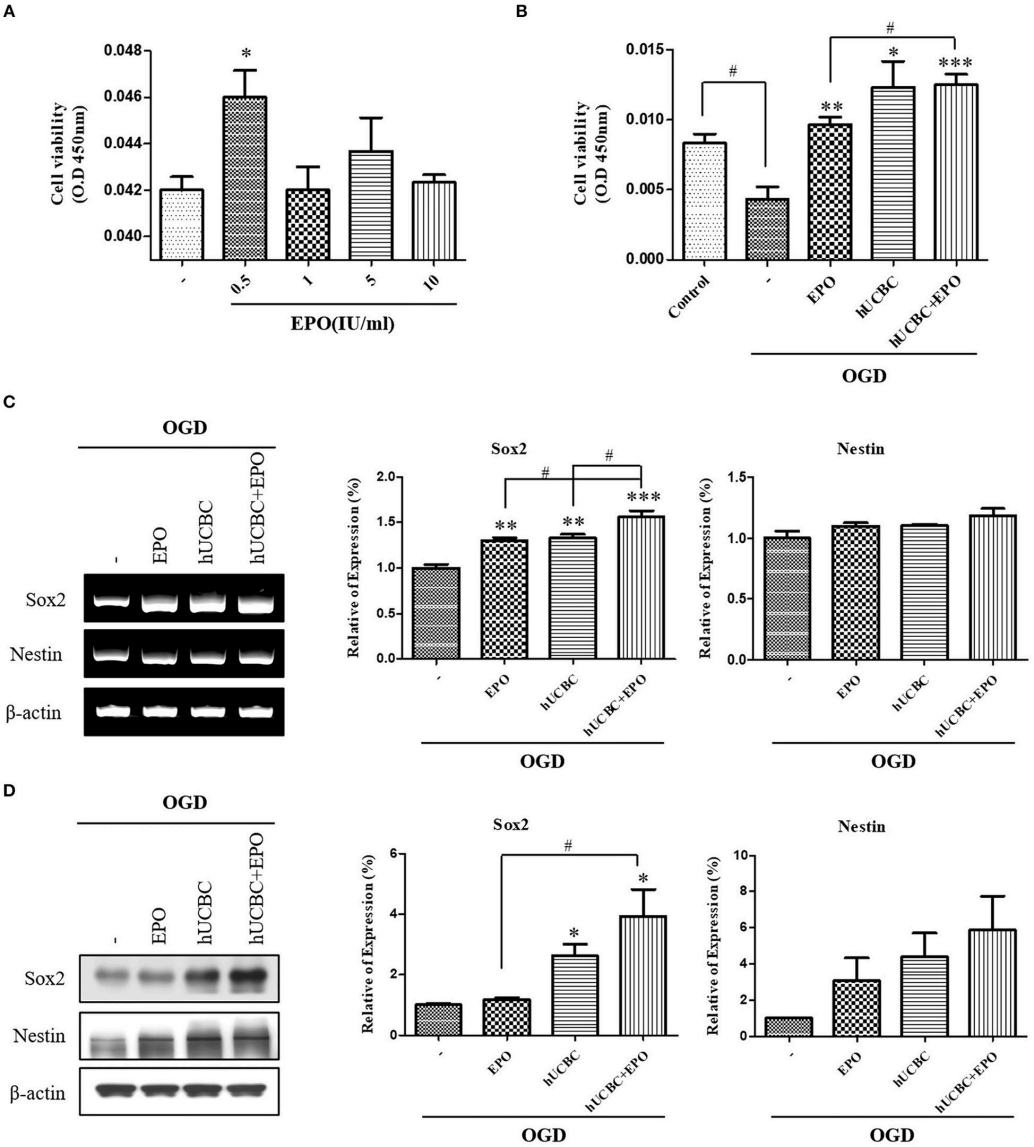
*In vitro* assays for neurogenesis following EPO, hUCBC and hUCBC+EPO treatment using OGD-injured neural stem cells. **(A)** Cell viability of EPO concentration in OGD-injured neural stem cells **(B)** cell viability was decreased by OGD, and increased by EPO and hUCBC and the most by hUCBC+EPO. **(C)** Reverse transcription polymerase chain reaction (RT-PCR) for expression of Sox2 and Nestin. Sox2 gene expression was significantly increased by EPO or hUCBC treatment, and it was most increased by hUCBC+EPO. **(D)** Western blotting for expression of Sox2 and Nestin. Sox2 was upregulated by hUCBC and hUCBC+EPO treatment. Data represent mean ± SEM. *N* = 3 per group. ^*^P < 0.05, ^**^P < 0.01, ^***^P < 0.005 vs. OGD-injured cells. ^#^*P* < 0.05 for inter-treatment effect comparison.

To evaluate the effect on neuronal proliferation in OGD-injured NSCs and to identify the appropriate concentration of culture media, NSCs were treated with 0, 0.5, 1, 5, or 10 IU/ml of EPO for 24 h after OGD (Figure 6A). A dose of 0.5 IU/ml of EPO was selected because it produced the best survival rate for NSCs. After OGD, WST assays were conducted; the results showed remarkable reduction in the viability of NSCs (Figure 6B; *P* < 0.05). The cell viability assay revealed the best survival rates in NSCs that received hUCBC and EPO combination treatment (*P* < 0.001), while hUCBC—(*P* = 0.05) or EPO—(*P* < 0.01) alone treatment also improved cell viability relative to OGD alone (Figure 6B). Next, we assessed Sox2, a neuronal proliferation marker, to evaluate the neurogenic effect of treatment with EPO, hUCBC, or hUCBC+EPO after OGD. Sox2 gene and protein expression were significantly upregulated in the hUCBC+EPO group (*P* < 0.05), while a similar response but to a lesser extent was seen for Nestin (*P* = 0.06) expression (Figures 6C,D).

### EPO, hUCBC, and hUCBC+EPO Enhanced Angiogenesis in OGD-Injured Endothelial Cell

**Figure 7:**
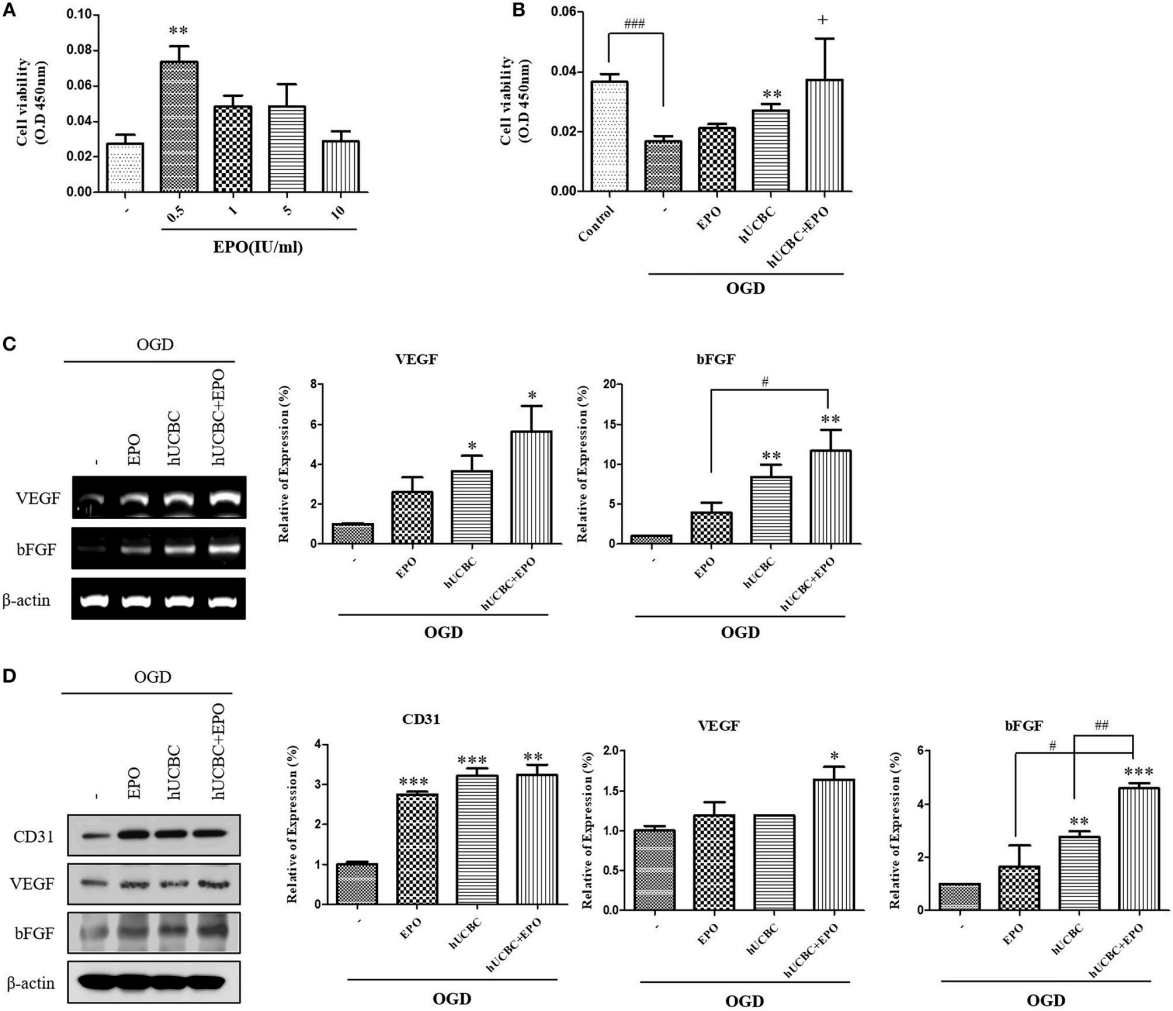
*In vitro* assays for angiogenesis following EPO, hUCBC and hUCBC+EPO treatment using OGD-injured bEnd.3 cells. **(A)** Cell viability of EPO concentration in OGD-injured bEnd.3 cells **(B)** Survival and cell viability were decreased by OGD, and increased by hUCBC and the most by hUCBC+EPO with marginal significance (+*P* = 0.057). **(C)** Reverse transcription polymerase chain reaction (RT-PCR) for expression of VEGF and bFGF. VEGF and bFGF gene expressions significantly increased by hUCBC treatment, and it was most increased by hUCBC+EPO. **(D)** Western blotting for expression of CD31, VEGF and bFGF. VEGF was upregulated by hUCBC+EPO. CD31 and bFGF was upregulated by hUCBC, and the most by hUCBC+EPO. Data represent mean ± SEM. *N* = 3 per group. ^*^P < 0.05, ^**^P < 0.01, ^***^P < 0.005 vs. OGD-injured cells. ^#^*P* < 0.05, ^##^*P* < 0.01, ^###^*P* < 0.005 for inter-treatment effect comparison.

Next, we administered EPO at the same dosages of 0, 0.5, 1, 5, or 10 IU/ml to bEnd.3 cells after OGD, and analyzed the results after 24 h (Figure 7A). Again, 0.5 IU/ml of EPO (*P* < 0.01) was selected because it yielded the best survival rate for bEnd.3 cells. After exposure to OGD for 24 h, viability was attenuated by ~50% as reported previously (Figure 7B). In OGD-injured bEnd.3 cells, treatment with hUCBC+EPO resulted in the best cell viability, while hUCBC-alone led to better viability than OGD injury without treatment (Figure 7B). According to an angiogenesis factor expression assay examining gene expressions of VEGF and bFGF, hUCBC+EPO (VEGF, *P* < 0.05; bFGF, *P* < 0.01) treatment resulted in highest levels of expression of both genes, while treatment with hUCBC—alone also increased expression of those genes after OGD (Figure 7C). Levels of the endothelial cell marker CD31 and the angiogenic protein markers VEGF and bFGF increased in the hUCBC+EPO group (CD31, *P* < 0.005; VEGF, *P* < 0.05; bFGF, *P* < 0.0001) relative to those following OGD injury without treatment (Figure 7D).

### Tube Formation Effect of EPO, hUCBC, and hUCBC+EPO in OGD in Endothelial Cell

**Figure 8:**
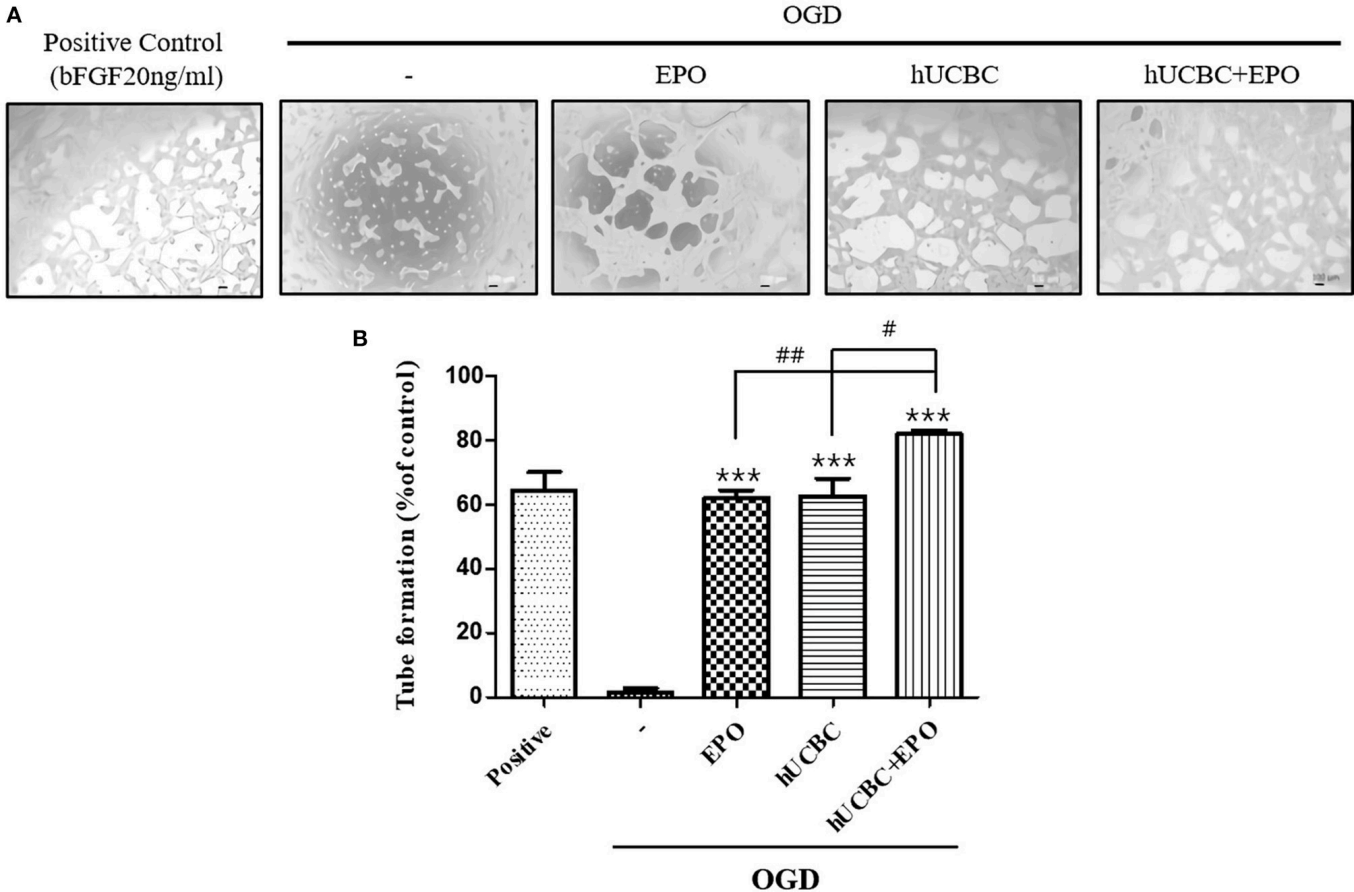
Tube formation assay by EPO, hUCBC and hUCBC+EPO treatment in OGD-induced bEnd.3 cells. **(A)** Images of tube formation in each groups: positive control was treated bFGF (20 ng/ml); control was not; co-cultured with EPO, hUCBC and hUCBC+EPO in OGD-induced cells. **(B)** Quantitative analysis of total number of tube formation. Total number of tubes were increased by EPO or hUCBC treatment, and the most by hUCBC+EPO. Data represent mean ± SEM. *N* = 3 per group. ^***^P < 0.005 vs. OGD-injured cells. ^#^*P* < 0.05, ^##^*P* < 0.01 for inter-treatment effect comparison.

Because tubular structure formation is a critical process in angiogenesis, we conducted a tube formation assay after OGD. As a result, the hUCBC+EPO (*P* < 0.0001) treatment remarkably increased the number of tubes, while treatment with either hUCBC- or EPO-alone also increased the number of tubes relative to OGD injury without treatment (Figure 8).

## Discussion

In this study, the efficacy of cell-based therapy in *in vivo* ischemic conditions was investigated. Post-ischemic treatment with EPO, hUCBC, or hUCBC+EPO improved functional outcomes in a subacute stroke rat model. In particular, hUCBC+EPO administration promoted functional recovery as measured using the mNSS and cylinder test better than treatment with EPO- or hUCBC-alone. Most previous studies tested the efficacy of cell-based therapy at 24 h after ischemic injury which reflects the acute stage of stroke (36, 37). The study is significant as it shows remarkable improvement in the behavioral test results of adult rats given hUCBC intravenously at 7 d after MCAO, a model representative of subacute stroke that was not been frequently studied (7). The current study also demonstrated the therapeutic efficacy of delayed administration of EPO during the subacute stage of stroke, which is in accordance with previous research (30). However, the effect of EPO seems to last only for a few days in this study. Furthermore, the efficacy of hUCBC administration appeared later than EPO and persisted. The combination of hUCBC and EPO showed a rapid initial response and long-lasting effect, from a week to 3 weeks after the therapy, and had the greatest efficacy of all the examined interventions. Therefore, these results indicate the possibility of overcoming issues regarding the weak efficacy of cell-based therapy in neurorestoration following stroke.

The therapeutic efficacy of hUCBC and EPO was also apparent in the brain tissue with manifestations of neurogenesis and angiogenesis in the present study. During the neurological recovery from stroke, both neurogenesis and angiogenesis occur (20, 22). Also in this study, neurogenesis and angiogenesis were enhanced together both *in vivo* and *in vitro* by treatment with EPO, hUCBC, or hUCBC+EPO. However, in terms of VEGF, its gene expression was elevated by hUCBC and hUCBC+EPO treatments (PCR), and its protein expression was elevated only by hUCBC+EPO treatments (western blotting) *in vitro*. The VEGF, a key angiogenic and permeability factor (24, 25), increases cerebral microvascular perfusion and induces neurological recovery when administered 48 h after ischemic stroke (38). VEGF is also likely to mediate the coupling of angiogenesis and neurogenesis after stroke (39). The results therefore indicate that only hUCBC+EPO treatment may induce the enhancement of VEGF expression, the most significant factor in neurogenesis and angiogenesis, even in later stage post-stroke.

The highest levels of neurogenesis and angiogenesis in the cortex occurred following hUCBC+EPO treatment; similar responses were seen for NSC and endothelial cells. Treatment with either hUCBC- or EPO-alone resulted in similar findings regarding neurogenesis and angiogenesis *in vivo* and *in vitro*, but to a lesser extent than that following hUCBC+EPO treatment. It was interesting that the number of NeuN(+) neuronal cells was increased by treatment with either hUCBC or EPO, but increased most following treatment with hUCBC+EPO. On the contrary, the number of GFAP(+) cells were decreased inversely by the treatments and hUCBC+EPO induced the greatest reduction. GFAP can be expressed by many cell types although astrocytes are the most representative and the role of astrocytes in maladaptive plasticity in the ischemic brain has been reported (40). Although astrocytes have two roles in the pathological mechanisms of neurological diseases (41), our finding of a reduction in the number of GFAP(+) cells can be interpreted as a reduction of astrogliosis. In relation to this reaction, it is notable that another glial maker, Iba-1(+) cells showed reversed reaction. Since this finding indicates more survival or activation of microglia by hUCBC or EPO treatment, role of the cells in neurogenesis can be inferred.

This study is not the first to illustrate the potential of cell-based therapy via combination with a growth factor. Because a study where brain-derived neurotrophic factor was administered alongside hBMSC in a stroke model showed positive results (42), variable approaches have been reported to enhance the neurorestorative effect of cell-based therapy (43). To present, reports have also revealed the therapeutic advantages of concomitant administration of other factors in cell-based therapy (44, 45). However, not all trials were successful. A recent study revealed potential side effects that are harmful in ischemic stroke when hBMSC were transplanted with granulocyte colony-stimulating factor, leading to findings of increased hemorrhagic transformation and astrogliosis with alterations in the blood-brain barrier (46). In the present study, the results seem to show an additive or synergistic effect of combined treatment with hUCBC and EPO, without a diluting effect, both for neurogenesis and angiogenesis. Furthermore, the finding of a reduction in astrogliosis was inversely correlated with the increase in neurogenesis and angiogenesis for all treatments among the groups, both *in vivo* and *in vitro*. Taken together, hUCBC and EPO appear to share the same signaling pathways in their therapeutic mechanisms for recovery after stroke. This therapeutic combination did not result in harmful adverse events, which is the most important point in clinical applications.

This study has certain limitations. First, the perfectly uniform allocation of the rats into four groups was not possible for all experimental subsets. However, this study was conducted following the principles of randomization. Second, the increase in the number of NeuN(+) cells in the cortex following treatment that was taken as reflective of neurogenesis can be the result of neuroprotection with increased survival of neuronal cells. We did not observe neuroregeneration *per se* after loss of neurons in the damaged area. Third, although the results in behavior, histological findings, and *in vitro* assays represent manifestations of neurogenesis and angiogenesis, more direct mechanisms that may explain the response of the host induced by hUCBC and EPO administration should be clarified. Also, this study did not reveal engraftment status of the intravenously administered cells. The authors presume that the treatments were given systemically, the response must be systemic and must have affected the brain. Considering their availability in clinical usage, it is difficult to give therapeutic cells directly to the brain, and because peripherally introduced cells demonstrated efficacy, the mechanism of action would be important to identify. In relation, further research on the blood-brain barrier might enlighten the therapeutic mechanism. Lastly, the common signaling pathway of hUCBC and EPO treatment for stroke injury should be elucidated.

In conclusion, our results suggest that hUCBC infusion in combination with EPO administration demonstrates therapeutic efficacy in the treatment of stroke-induced injury by promoting neurogenesis and angiogenesis. Further research that delineates the therapeutic mechanism of systemically administered hUCBC and EPO is required.

## Ethics Statement

All experimental procedures involving animals were performed in accordance with the Guide for the Care and Use of Laboratory Animals as adopted and promulgated by the U. S. National Institutes of Health and were approved by CHA University Institutional Animal Care & Use Committee (IACUC150018, IACUC180018, IACUC180181).

## Author Contributions

SH conducted animal experiments, analyzed the data, and drafted the manuscript. JC performed *in vitro* assays and analyzed the data. MK designed and conceptualized this study, interpreted the results, and revised the manuscript.

### Conflict of Interest Statement

The authors declare that the research was conducted in the absence of any commercial or financial relationships that could be construed as a potential conflict of interest.
